# Ferric quinate (QPLEX) inhibits the interaction of major outer membrane protein (MOMP) with the Lewis b (Le^b^) antigen and limits *Campylobacter* colonization in broilers

**DOI:** 10.3389/fmicb.2023.1146418

**Published:** 2023-03-10

**Authors:** Jennifer C. Okoye, Alexandria Holland, Matthaios Pitoulias, Vasileios Paschalis, Artem Piddubnyi, Osman A. Dufailu, Thomas Borén, Neil J. Oldfield, Jafar Mahdavi, Panos Soultanas

**Affiliations:** ^1^Biodiscovery Institute, School of Chemistry, University of Nottingham, Nottingham, United Kingdom; ^2^Department Medical Biochemistry and Biophysics, Umeå University, Umeå, Sweden; ^3^SUMEYA, The Ukrainian-Swedish Research Center, Sumy State University, Sumy, Ukraine; ^4^Faculty of Engineering and Science, School of Science, University of Greenwich, London, United Kingdom; ^5^School of Life Sciences, University of Nottingham, Nottingham, United Kingdom

**Keywords:** major outer membrane protein (MOMP), *Campylobacter*, broilers, iron chelates, Lewis b (Le^b^) antigen, gastrointestinal

## Abstract

*Campylobacter jejuni* colonizes hosts by interacting with Blood Group Antigens (BgAgs) on the surface of gastrointestinal epithelia. Genetic variations in BgAg expression affects host susceptibility to *C. jejuni*. Here, we show that the essential major outer membrane protein (MOMP) of *C. jejuni* NCTC11168 binds to the Lewis b (Le^b^) antigen on the gastrointestinal epithelia of host tissues and this interaction can be competitively inhibited by ferric quinate (QPLEX), a ferric chelate structurally similar to bacterial siderophores. We provide evidence that QPLEX competitively inhibits the MOMP-Le^b^ interaction. Furthermore, we demonstrate that QPLEX can be used as a feed additive in broiler farming to significantly reduce *C. jejuni* colonization. Our results indicate that QPLEX can be a viable alternative to the preventative use of antibiotics in broiler farming to combat *C. jejuni* infections.

## 1. Introduction

Genetic blood group antigen (BgAgs) phenotypes among individuals and populations affect host susceptibility to bacterial infections (de Mattos, 2016). The ABO blood group system, secretor system (encoded by the *FUT3* gene) and histo-blood group systems control the expression of most carbohydrate structures present in areas of the body occupied by microorganisms (Henry, 2001). The presentation of these carbohydrates on cells offers potential attachment receptors for pathogenic and non-pathogenic microorganisms, influencing resistance to infection or resulting illness (Anstee, 2010). ABO, H, secretor and Lewis BgAg carbohydrates are synthesized by specific glycosyltransferases, encoded by the *ABO*, *FUT1*, *FUT2*, and *FUT3* genes, that incorporate monosaccharide units sequentially to linear or branched precursor oligosaccharide chains, thus modifying and creating new antigenic specificities (Oriol, 1995; King et al., 2000). Selective pressure imposed by disease-causing microorganisms contributes to the variation seen amongst populations (Roberts, 1984; Henry et al., 1995; Oriol, 1995). The variability resulting from the BgAg systems has important implications in susceptibility to infections, innate and adaptive immune responses, cancer and solid organ transplantation. For example, individuals with the O blood group are more prone to gastroduodenal diseases, such as gastritis and peptic ulcers, because *Helicobacter pylori* strains expressing the blood group antigen binding adhesin (BabA) are able to bind to the Le^b^ carbohydrate that is highly expressed in gastric epithelial cells and related to O and secretor BgAgs. It was also observed that the South American specialist strains of *H. pylori* showed higher binding to H type 1 and Le^b^ carbohydrates than generalist strains which correlated with the predominance of the O blood group in American Indians (Ilver et al., 1998; Aspholm-Hurtig et al., 2004). Furthermore, the H type 2 carbohydrate inhibits the attachment of *Campylobacter jejuni* to intestinal cells thereby protecting breastfed babies from *Campylobacter* (Ruiz-Palacios et al., 2003).

Bacterial adhesion is mediated through specific interactions between bacterial outer membrane proteins, called adhesins, and structures expressed on the surface of host cells that serve as cognate adhesin receptors. These receptors often carry oligosaccharide (glycan) modifications. Several proteins on *C. jejuni* contribute to its adherence to eukaryotic cells, one of which is the major outer membrane protein (MOMP). *Campylobacter* adheres to the intestinal mucosa through the epithelial cell surface H blood group (fucosyl α1,2) epitopes and invades host cells. Some *Campylobacter* strains bound to several related fucose-containing BgAgs but bound most strongly to H ligands (Ruiz-Palacios et al., 2003). Using ELISA binding studies, *C. jejuni* was shown to bind to BgAgs core-I, core-II, H-II, Le^b^, Ley, and Lex BgAgs. Retagging was used to identify the *C. jejuni* BgAg-binding adhesins as the MOMP and flagellar protein, FlaA (Mahdavi et al., 2014). MOMP was also shown to bind to fibronectin and INT 407 cell membranes (Moser et al., 1997). MOMP is a trimeric 18-stranded antiparallel β-barrel porin with an elliptical shape typical of 18-stranded porins and resembling the structure of the *H. pylori* adhesin BabA (Subedi et al., 2014; Ferrara et al., 2016). It contains an *N*-terminal extracellular host-binding domain and a C-terminal outer membrane–spanning domain predicted to form a β-barrel structure similar to that of porins. *C. jejuni* NCTC11168 MOMP was shown to be *O*-glycosylated at T268 and the *O*-linked glycan was instrumental for MOMP-mediated adhesion to BgAgs and chicken colonization (Mahdavi et al., 2014). Deletion of *pseD*, which encodes a putative PseAm transferase, resulted in a significant reduction in the binding of *C. jejuni* NCTC11168 to the BgAgs, suggesting that PseD is required for *O*-glycosylation of MOMP (Mahdavi et al., 2014). A single T268G mutation in MOMP abolished the ability of *C. jejuni* NCTC11168 to colonize and infect broilers, thus confirming the importance of this primary adhesin for attachment to the gut epithelia of chickens in order to establish infection (Mahdavi et al., 2014).

*Campylobacter spp* are present in the gastrointestinal flora of many birds but chickens constitute the most important source of human infection (Jacobs-Reitsma, 1997). *Campylobacter jejuni* typically colonize the avian gastrointestinal tract to a high level (9 log_10_CFU g^–1^ contents). It is usually found at the greatest levels in the mucosal crypts of the caeca, and it can be recovered at lower levels from other sites including the crop, liver, gizzard, small intestine and cloaca (Beery et al., 1988). Prevention and treatment of *Campylobacter* infections in humans are hampered by our poor understanding of the underpinning molecular interactions between the pathogen and hosts such as human and chicken. Also, the role of host cell surface glycoconjugates, such as BgAgs, in *Campylobacter* colonization and invasion is largely unknown. Recent studies showed that ferric tyrosinate (TYPLEX), a ferric chelate structurally resembling bacterial siderophores, when used as feed additive protected commercial broilers from *C. jejuni* infection reducing loads by 2–3 log_10_ (Currie et al., 2018; Khattak et al., 2018; Skoufos et al., 2019). This effect was postulated to be through binding of TYPLEX to MOMP, preventing adhesion of *C. jejuni* to the gut epithelia of broilers but no experimental evidence is available to support this hypothesis (Khattak et al., 2018). Another ferric chelate, ferric quinate (QPLEX) which is structurally similar to TYPLEX, was recently shown to interact directly with MOMP and to translocate through the porin into the periplasmic space (Okoye et al., 2022). Here, we show that QPLEX competitively inhibits binding of MOMP to BgAgs, including Le^b^. In a similar manner to TYPLEX, QPLEX is also shown to protect commercial broilers from *C. jejuni* infection when used as feed additive. Collectively, our data indicate that ferric chelates such as QPLEX and TYPLEX offer an alternative to preventative use of antibiotic treatment in commercial broiler farming thus helping to reduce the spread of antibiotic resistance through animal husbandry.

## 2. Materials and methods

### 2.1. Protein purifications, conjugation with fluorescent probes and synthesis of QPLEX

Native MOMP, MOMP^T/G268^, and MOMP^ΔpseD^ were purified directly from the NCTC11168 wild type, *T/G268* and Δ*pseD C. jejuni* strains (Mahdavi et al., 2014), as described elsewhere (Okoye et al., 2022). Native NCTC11168 MOMP was conjugated to fluorescein (NHS-Fluorescein, Thermo Scientific) and far-red Alexa Fluor™ 647 (NHS Ester, Thermo Scientific) via the dyes’ succinymidyl ester to form stable amide bonds with primary amines on MOMP’s surface. Native MOMP was diluted to 1 mg/ml in 1 ml PBS. 15 mmol molar excess of the fluorescent probe to MOMP was used to conjugate the probe to the protein (4 μl of the fluorescent probe solution, prepared by dissolving 1 mg of the probe in 100 μl DMSO). The solution was mixed well and incubated on ice for 2 h. Non-reacted fluorescent probe was removed by dialysis using Slide-A-Lyzer Dialysis Cassettes, 10,000 MWCO, for volumes up to 2 ml. Successful labeling was confirmed by running the conjugated proteins on an sodium dodecyl sulfate (SDS) PAGE gel and visualizing it under UV light. All MOMP proteins were confirmed by Western Blot using an anti-MOMP rabbit antibody (Supplementary Figure 7A see Mahdavi et al., 2014 for the source of the rabbit anti-MOMP antibody).

SDS PAGE analysis of the purified MOMP, MOMP^T/G268^ and MOMP^ΔpseD^ proteins revealed different electrophoretic mobilities, indicative of lack of *O*-glycosylation in the MOMP^T/G268^ and MOMP^ΔpseD^ proteins compared to the native MOMP [Supplementary Figure 7B and (Mahdavi et al., 2014)].

Ferric quinate was synthesized by mixing quinic acid and ferric chloride in a 3:1 M ratio and subsequent crystallization, as described elsewhere (Menelaou et al., 2009; Okoye et al., 2022).

### 2.2. The FVB/N-Le^b^ mice and preparation of tissue sections

All animals and experiments in this study were approved by the ethics committee at Umeå University (ethical permit Dnr. A10-2018, A19-18) and complied with the regulations and rules of the Swedish Animal Welfare Agency and with the European Communities’ Council Directive of 22.09.2010 (2010/63/EU). *FVB/N* transgenic mice that express human α-1,3/4-fucosyltransferase and thus have Le^b^-glycosylated gastric epithelium (Falk et al., 1995; Fagerberg et al., 2009) were used for this study. All breeder pairs were tested positive for transgenicity by PCR. The mice were maintained by trained personnel at the animal facility of the Umeå Center for Comparative Biology (UCCB) under pathogen-free conditions. Mice were housed in a 12-h dark/light cycle environment with *ad libitum* access to food and tap water.

Mice were sacrificed by cervical dislocation, and their stomachs were dissected through the small curvature. A representative part of the organ with all anatomical regions (forestomach, corpus, and antrum) was placed in a standard histological cassette (Thermo Fisher Scientific, USA) between two biopsy pads (Thermo Fisher Scientific, USA) to prevent tissue deformation. Tissue samples were fixed in a 4% v/v neutral paraformaldehyde aqueous solution (HistoLab, Sweden) for 24 h and saturated with paraffin in a Leica ASP300S tissue processor (Leica Microsystems, Germany). Standard paraffin blocks were made with an embedding station Leica EG1140 (Leica Microsystems, Germany) with Histowax paraffin (HistoLab, Sweden). Sections with a thickness of 4 μm were cut with a Leica^®^ RM2255 automated microtome (Leica Microsystems, Germany), placed on SuperFrost Plus™ adhesion slides (Thermo Fisher Scientific, Waltham, MA, USA), and dried overnight at + 37°C. Human tissue was obtained from biopsy specimens of normal (non-*C. jejuni* infected, non-inflamed) human corpus, antrum, duodenum and colon were collected from human biopsies, as part of diagnoses for gastrointestinal complaints.

### 2.3. Immunofluorescent imaging: Preparation of tissue sections

Human tissue sections mounted on glass slides were from biopsy specimens of normal (non-*C. jejuni* infected, non-inflamed) human corpus, antrum, duodenum and colon. Mouse tissues were from normal mouse (not expressing Le^b^) and a transgenic mouse model (based on FVB/N, expressing Le^b^), (Falk et al., 1995). Specimens were fixed in PBS, 4% w/v paraformaldehyde solution for 4 h, then processed in paraffin blocks and sectioned according to standard procedures. The sections were deparaffinized by soaking in xylene and rehydrated by immersing the slides through serial dilutions of ethanol. Firstly, the sections were immersed in xylene, for three washes of 5 min each, followed by 2 washes of 5 min each in 100% v/v ethanol, two washes 5 min each in 95% v/v ethanol, two washes 5 min each in 70% v/v ethanol, two washes 5 min each in 50% v/v ethanol and a last wash in deionized water for 10 min.

### 2.4. Immunofluorescent imaging: Autofluorescence quenching

Vector TrueVIEW Autofluorescence Quenching Kit (Fisher Scientific) was used to remove non-specific background autofluorescence in tissue sections due to aldehyde fixation, red-blood cells, and structural elements such as collagen and elastin. Firstly, the extent of autofluorescence with negative control unstained sections was determined in each color channel. The reagents were prepared as specified in the Vector^®^ TrueVIEW^®^ Autofluorescence Quenching Kit user manual. Excess water was drained from tissue sections and a circle was drawn on the slide around the tissue with a hydrophobic barrier pen. Tissue sections were then completely covered with 150 μl of reagent and incubated for 2–5 min. After incubation, sections were washed in PBS for 5 min and excess buffer was drained before mounting the tissues with coverslips. Treatment of tissue sections for autofluorescence quenching turned them blue but this did not interfere with visualization. Optimization studies were carried out using human tissue slides, as described in the SI information (Supplementary Figures 1–3).

### 2.5. Immunofluorescent imaging: Staining with fluorescently conjugated MOMP

Deparaffinized, rehydrated and autofluorescence quenched sections were incubated in 100 μl of 7 p.m. to 7 μM of Alexa Fluor™ 647 or fluorescein labeled native NCTC11168 MOMP protein in PBS. The concentration of MOMP was increased from 7 p.m. to 7 μM until protein staining could be seen. After washing in PBS-0.5% v/v Tween 3 times for 5 min the slides were incubated and quenched in 100 μl Vector TrueVIEW Autofluorescence Quenching Kit and fluorescent staining was visualized by confocal microscopy and images analyzed by ImageJ.

### 2.6. Immunofluorescent imaging: Staining with native MOMP

Deparaffinized and rehydrated tissue sections were incubated in 100 μl of 5 μM of native NCTC11168 MOMP in blocking buffer of PBS and 0.1% w/v BSA for 1 h at room temperature. The slides were then washed with PBS-0.5% v/v Tween, 3 times for 5 min before incubating in 100 μl 1:500 rabbit anti-MOMP antibody in blocking buffer of PBS and 0.1% w/v BSA for 1 h at room temperature. After two further wash steps, the slides were incubated in 100 μl Alexa fluor 633 conjugated goat anti-rabbit secondary antibody (Thermo Fisher #A-21070). After three wash steps, the slides were incubated and quenched in 100 μl Vector TrueVIEW Autofluorescence Quenching Kit and fluorescent staining was visualized by confocal microscopy and images analyzed by ImageJ.

### 2.7. Immunofluorescent imaging: Staining with Le^b^

Deparaffinized and rehydrated sections were incubated in 100 μL of 1:500 dilution of anti-Le^b^ (Seraclone Anti-B #801350) in blocking buffer of PBS and 0.1% w/v BSA for 1 h at room temperature. The slides were then washed with PBS-0.5% v/v Tween, 3 times for 5 min before incubating with 100 μl 1:500 dilution of goat anti-mouse IgG antibody conjugated to Alexa Fluor 633 (Thermo Fisher # A-21052) in blocking buffer for 1 h at room temperature. After three wash steps, the slides were incubated and quenched in 100 μl Vector TrueVIEW Autofluorescence Quenching Kit and fluorescent staining was visualized by confocal microscopy and images analyzed by ImageJ. In competition experiments increasing concentrations of non-conjugated Le^b^-hexasaccharide (IsoSep AB, Sweden) was used.

### 2.8. Confocal microscopy

Tissue slides were visualized with Zeiss LSM 880 confocal microscope, using 20×/0.8 NA objective. Lasers, 488 and 633 nm, respectively, were used to visualize residual autofluorescence and MOMP staining. The 633 nm laser was used to create transmitted brightfield images as third channel. Detection (emission detected) was set to 499–517 nm for the green and 640–735 nm for the far-red, respectively. Zen Black software and Image j Fiji software were used to acquire images and perform image processing and analysis. 16-bit raw images of the far-red were visualized on a scale of low and high values, as noted under each image, in the Brightness Contrast tool of Fiji. Fluorescence intensity was quantified by subtracting background fluorescence from total fluorescence. A tissue area was viewed in the far-red channel and outlined with the freehand region of interest tool on ImageJ. Desired measurement parameters were set by going to Analyze > Set Measurements and selecting Mean Gray Value. The selected area was then analyzed by right clicking the image then Analyze > Measure. The measurement data from the pop-up window was copied into a spreadsheet (GraphPad Prism). The background signal was obtained by repeating the same process for the same selected area but viewed in the green channel, which determines the autofluorescence. This was repeated for 20 slides per QPLEX concentration. The total fluorescence corrected for background in units of mean gray values was obtained by subtracting the signal from the green channel from the total signal in the far-red channel and the mean gray values were plotted in GraphPad Prism as a function of QPLEX concentration.

### 2.9. Digoxigenin labeling of bacteria

Colonies of bacteria were suspended in sodium carbonate buffer and washed three times with the same buffer. OD_600_ was adjusted to 1.0, followed by addition of 1 μl of digoxigenin 10 μg/μl (Pierce, UK) and incubating for 1 h on shaker at room temperature. After 1 h, cells were washed three more times with PBS-T. The suspension was made up with 1% BSA-PBS when adjusting to a specific OD_600_ required for ELISA. Freshly labeled bacteria were used each time in the ELISA experiments in the presence or absence of QPLEX (34 μM).

### 2.10. Quantification of fluorescence intensity

Fluorescence intensity was quantified by subtracting from the total fluorescence the background fluorescence. Using ImageJ, the tissue area in the far-red channel was outlined with the free-hand region of interest tool. Measurement parameters were set in Analyze > Set Measurements and selecting the Mean Gray Value. The selected area was analyzed in Analyze > Measure and the measurements data were copied into a spreadsheet. To obtain a measurement for the background fluorescence, the process was repeated for the exact same selected area in the green fluorescence channel. This procedure was repeated for 20 separate tissue slides per QPLEX or Le^b^ concentration. The corrected total fluorescence in units of mean gray value were plotted for each concentration of QPLEX or Le^b^ in GraphPad Prism.

### 2.11. ELISA

All solutions used in ELISA were made up in 1% w/v BSA in PBS and all incubations were carried out at room temperature on the shaker unless otherwise stated. PBS-T was used for all washing steps. Coupling of BSA-conjugated BgAgs (pentameric sugars from IsoSep AB, Sweden) to 96 well plates (NUNC Immobilizer Amino) was carried out by adding 100 μl of a 5 μg/ml sugar solution in sodium carbonate buffer to each well. Control wells contained BSA in PBS buffer at 100 μg/ml. Plates were incubated for 2 h before removing the solutions and washing three times. All wells were blocked by addition of 100 μl of 100 μg/ml BSA in PBS buffer and incubation, as before. Plates were emptied and tapped dry. Digoxigenin tagged bacteria were diluted to an OD_600_ of 0.05 and pre-incubated with QPLEX (34 μM) or not (control). 100 μl of bacterial solution was added to each well and plates were incubated overnight. Plates were washed three times (ELISA-washer was used for three washes with 1 min interval) before adding 100 μl anti-digoxigenin-POD solution (1 in 5,000, Roche Diagnostics) to each well and incubating for 1 h. Plates were washed five times and tapped dry before developing in ABTS solution (Roche Diagnostics) and were read at 405 nm. Specific binding was determined by subtracting the binding to BSA from the binding to the BgAgs for each strain.

### 2.12. Statistical analysis for ELISA

Multiple *t* tests, one-way analysis of variance (ANOVA), and paired/unpaired *t* tests were performed on the relevant data sets. The one-way analysis of variance (ANOVA) was used to determine whether there were any statistically significant differences between the means of three or more independent (unrelated) groups. ANOVA was applied to data obtained to analyses the statistically significant difference between binding of native NCTC11168 MOMP, MOMP^T/G268^, and MOMP^ΔpseD^ to Le^b^. The same analysis was applied to measure statistically significant differences between binding of Le^b^ to MOMP in the absence and presence of QPLEX. For increased statistical power, ANOVA was used when comparing across data sets with three or more groups as ANOVA tests consider the variation between the means and within each mean.

### 2.13. Broiler study

The efficacy of QPLEX as a feed additive was assessed in broiler studies conducted at the Roslin Institute under the Unique Study Code: RGT136/2016 in compliance with current quality standards for EU feed additive applications. Procedures, documentation, records and equipment were checked and calibrated, as relevant, in order to assure that the study was conducted in accordance with the regulations, the specified protocol and relevant Standard Operating Procedures (see Supplementary Information, Supplementary Tables 1–9 for details).

### 2.14. Specific plates used for *Campylobacter spp*. determination and quantification

Gut, caeca and litter samples were collected and sent on dry ice for microbiological examination at Nottingham University. A sterile scalpel was used to cut off the blind end of both caeca sacks from each broiler. For each sample, half gram of content from each caeca sack, in total 1 g, was weighed out into sterile Universal bottles. 2 ml of sterile Maximum Recovery Diluent (MRD, R60796 Thermo-Fisher) was added to each container and mixed thoroughly. This constituted the 1:2 (w/v) dilution. These aliquots were then further serially diluted (e.g., 60 μl sample + 240 μl of MRD solution), 5, 25, 125, 625, and 3,125 in MRD to give different dilutions. 5 μl of each of the dilutions (a factor of 400; 2,000 μL original sample/5 μL) was then spotted on *Campylobacter spp*. selective blood agar (CCDA; Thermo-Fisher, PO0119A) and *Campylobacter spp*. chromogenic Brilliance CampyCount (Thermo-Fisher, PO1185A) agar plates. Plates were incubated under microaerophilic conditions at 42°C for 48 h. Following incubation, plates were assessed for the presence or absence of *Campylobacter spp*. In addition, plates cultured with appropriate dilutions were selected and colonies were enumerated.

### 2.15. Additional tests

As a confirmatory measurement, five colonies/treatment from 6 presumptively positive plates were selected and sub-cultured onto paired blood agar plates (Oxoid PB0114). These plates were incubated, respectively, at 37°C for 48 h aerobically/microaerobically. The presence of *Campylobacter* was indicated by lack of growth aerobically and morphologically consistent growth micro-aerobically. In addition to this, Gram stains were performed on all presumptively positive samples. As a further step, oxidase strips (Oxoid MB0266) were used to confirm that the samples were oxidase positive.

### 2.16. Statistical analysis of data for *Campylobacter* spp. quantification

The data obtained were subjected to *t*-test (unpaired, two tailed) analysis of variance to determine the effect of experimental diets on broiler bacterial load i.e., *Campylobacter spp.*, using a Prism 10 statistical software package. The bacterial counts were as actual number prior to analysis. All statements of significance are based on the probability level of *p* ≤ 0.05, (≤ 0.0001****, ≤ 0.001***, ≤ 0.01**, ≤ 0.1*). The following orthogonal contrast were used to compare specific treatments: Contrast 1 (CT vs. 0.22 g/kg QPLEX): To compare positive control group (CT) with 0.22 g/kg QPLEX-treated group.

## 3. Results

### 3.1. *Ex vivo* analyses of MOMP binding to stomach tissue from transgenic mouse expressing Le^b^

**FIGURE 1:**
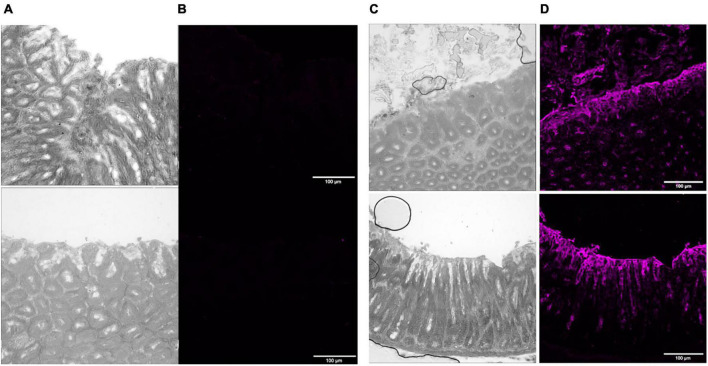
P28 FVB/N mouse stomach normal versus transgenic Le^b^ + ve tissue treated with Le^b^ specific mouse antibody, followed by staining with far-red with goat anti-mouse secondary antibody and an auto-fluorescence quencher. Images are magnified 20× to focus on different parts of the tissue sections. Panels **(A,C)** show light phase images at optimum contrast to display corresponding normal and transgenic Le^b^ + ve tissue structure, respectively. Panels **(B,D)** show images from normal and transgenic Le^b^ + ve tissues, respectively, taken in the far-red fluorescent channel on a scale of 54 low and 6,185 high values in the Brightness Contrast tool of Fiji, and modified to magenta for easier viewing. Scale bars represent 100 μm. The images shown here are representative of a wider set.

**FIGURE 2:**
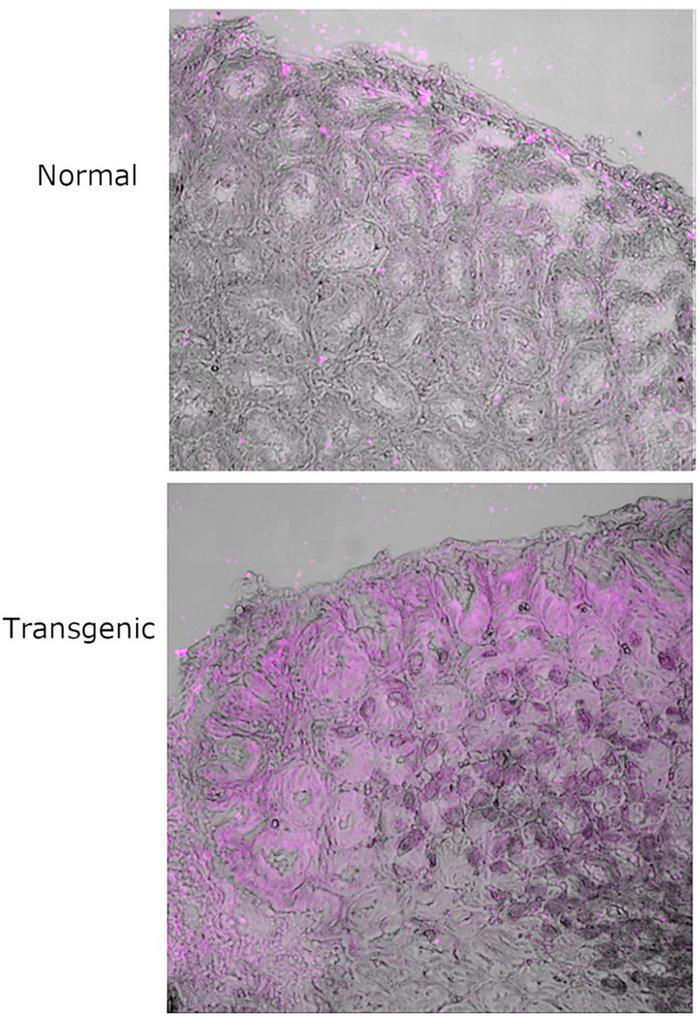
20 × magnification of two regions of normal and Le^b^ positive transgenic P28 FVB/N mouse stomach tissue treated with native MOMP followed by detection with anti-MOMP rabbit antibody and fluorescent-labeled secondary antibody and auto-fluorescence quencher. Light phase images have been overlaid with the fluorescent far-red channel images. Images in far-red channel visualized on a scale of 0 low and 65,279 high values in the Brightness Contrast tool and pictured in magenta. The images shown here are representative of a wider set.

*Ex vivo* immunofluorescence imaging optimization studies to eliminate autofluorescence were carried out with human tissue sections (Supplementary Figures 1–3) because of a limited number of chicken tissue sections which were needed for the main focus of this work. To investigate the MOMP-Le^b^ interaction, we used tissue sections from a transgenic mouse model (based on FVB/N). These mice do not express Le^b^ in any of their stomach or intestinal epithelial cell lineages, but genetically modified FVB/N mice have been produced that express Le^b^ (Falk et al., 1995). Tissue sections were obtained from the stomach, including the forestomach, corpus, pyloric part and the initial part of the duodenum. Comparative studies of normal and transgenic FVB/N stomach tissue sections stained with anti-Le^b^ specific antibody confirmed the expression of Le^b^ on the stomach epithelium of transgenic mice and not in normal mice (Figure 1). Similar studies carried out with anti-MOMP antibody, revealed substantially more binding of native MOMP in stomach tissue sections from the Le^b^ over-expressing FVB/N transgenic mice compared to normal mice (Figure 2). Some observed binding of MOMP to tissue slides from normal mice is likely because of the interaction of MOMP with epithelial ligands other than Le^b^, but in the transgenic mice binding is substantially more, as MOMP binds additionally and specifically to expressed Le^b^ on the stomach epithelium of these mice.

**FIGURE 3:**
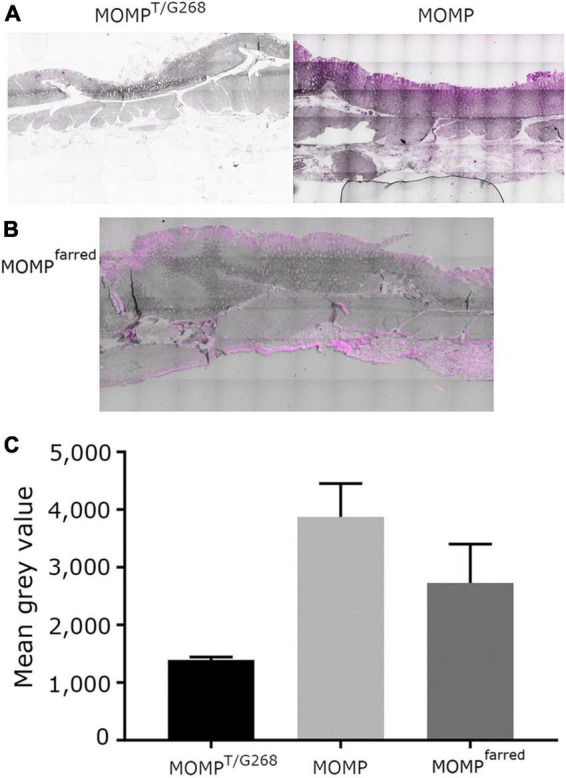
**(A)** FVB/N Le^b^ positive transgenic stomach tissue treated with either MOMP^T/G268^ or native MOMP as indicated, then by anti-MOMP rabbit antibody detected with far-red tagged secondary anti-rabbit antibody and auto-fluorescence quencher. **(B)** FVB/N Le^b^ positive transgenic stomach tissue treated with MOMP^farred^. In all cases, light phase images have been overlaid with the fluorescent far-red channel images. All images visualized on a scale of 0 low and 65,279 high values in the Brightness Contrast tool of Fiji in the far-red channel and converted to magenta for easier viewing. Scale bars represent 500 μm. The images shown here are representative of a wider set. **(C)** Quantification of signal emitted from staining treatments, as described in panels **(A,B)**. The amount of signal from fluorescence in the red channel was normalized against background fluorescence measured in the green channel. The bar graphs represent the mean and S.E.M. of the fluorescence signals of four separate images per slide treatment, in terms of mean gray values.

**FIGURE 4:**
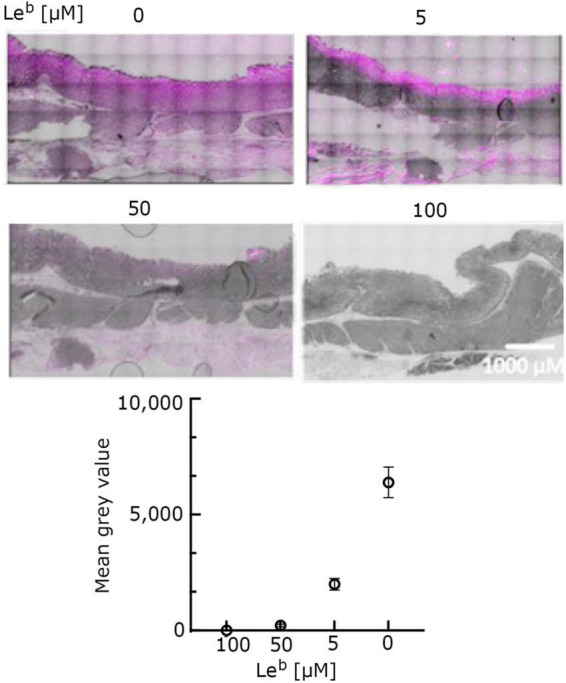
Le^b^ + ve transgenic FVB/N mouse stomach tissue incubated with native MOMP (5 μM) and increasing concentrations of non-conjugated Le^b^-hexasaccharide (5, 50, and 100 μM, as indicated) for 1 h at room temperature, in a competitive binding study. Tissue slides were stained with anti-MOMP rabbit antibody and counterstained with far-red tagged goat anti-rabbit antibody and auto-fluorescence quencher. Representative images from a bigger set of images are shown here. Images in far-red channel visualized on a scale of 512 low and 11,520 high values in the Brightness Contrast tool of Fiji and converted to magenta for easier viewing. The images shown here are representative of a wider set. Quantification of fluorescence (corrected for background) is reported as total fluorescence of mean gray values observed at each Le^b^ concentration (*n* ≥ 3).

*O*-glycosylation at T268 has been shown before to be important for the interaction of MOMP with Le^b^, as MOMP purified from the NCTC11168MOMP^T/G268^ and NCTC11168Δ*pseD C. jejuni* strains exhibited reduced binding to Le^b^, indicating that PseD is required for glycosylation at the T268 position (Mahdavi et al., 2014 and Supplementary Figure 4). To confirm that increased MOMP binding observed in the FVB/N transgenic mice tissues is specific to MOMP binding to Le^b^, we carried out immunofluorescence experiments with MOMP^T/G268^ (native MOMP carrying the T268G mutation) and MOMP^farred^ (native MOMP conjugated to far-red Alexa Fluor ™ 647). Our data suggested that MOMP^T/G268^ exhibited less binding to FVB/N transgenic stomach tissue compared to native MOMP (Figure 3A). Direct binding of MOMP^farred^ to FVB/N transgenic stomach tissue further confirmed the specificity of the MOMP-Le^b^ interaction (Figures 3B, C). Finally, binding of native MOMP to FVB/N transgenic stomach tissue was challenged with increasing concentrations (0.5, 5, and 50 μM) of soluble Le^b^ in a competition binding study. We observed a reduction of MOMP binding to FVB/N transgenic stomach tissue slices as the concentration of soluble Le^b^ increased, further confirming the specificity of the MOMP-Le^b^ interaction in our experimental system (Figure 4).

### 3.2. Inhibition of MOMP binding to BgAgs by QPLEX

**FIGURE 5:**
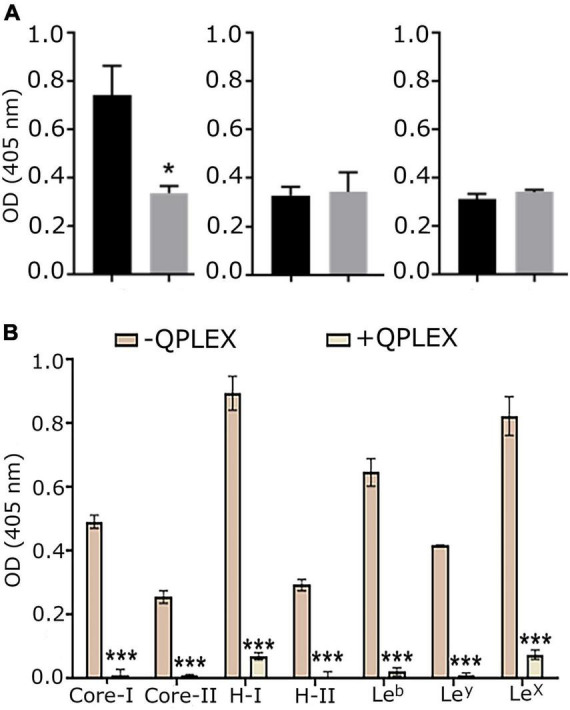
QPLEX inhibits binding of MOMP to BgAgs. **(A)** Binding of native MOMP (left), MOMP^T/G268^ (middle) and MOMP^Δ pseD^ (right) at 1 p.m. to non-conjugated Le^b^-hexasaccharide (1 μM adsorbed onto the well surfaces of Nunc Maxisorp plates) in ELISA in the absence (black bars) presence (gray bars) of QPLEX (1 nM). This was followed by incubating with primary antibody against MOMP and alkaline phosphatase (AP) conjugated secondary antibody. The Tecan was used to measure absorbance at 405 nm by the AP substrate. The results show the maximum absorbance obtained during 16 h assays after subtracting the absorbance values from BSA negative controls. Standard error bars about the means from triplicate experiments are shown. The significant differences between binding of Le^b^ to MOMP, MOMP^T/G268^ and MOMP^Δ pseD^ proteins were calculated using ANOVA statistical test (* = *p* < 0.5). **(B)** The competitive effect of soluble glycans conjugated to BSA (Core-I, Core-II, H-I, H-II, Le^b^, Le^y^, and Le^X^) on the attachment of *Campylobacter jejuni* NCTC11168 to a series of BgAgs was investigated. ELISA plate was coated with a selection of six BgAgs. Specific binding was calculated by subtracting the BSA (negative control) values from the BgAg absorbance. Binding of *C. jejuni* NCTC11168 to BgAgs was inhibited significantly (*** = *p* < 0.005) by pre-incubation of cells with QPLEX (34 μM), prior to adding to the ELISA plate. Untreated bacteria were used as a control and that was defined as 100% binding reference. Student’s *t-test* was used to assess the significance of differences between means in non-inhibited binding and inhibition analyses. Error bars = mean of triplicate values at two occasions ± SED (*n* = 3).

**FIGURE 6:**
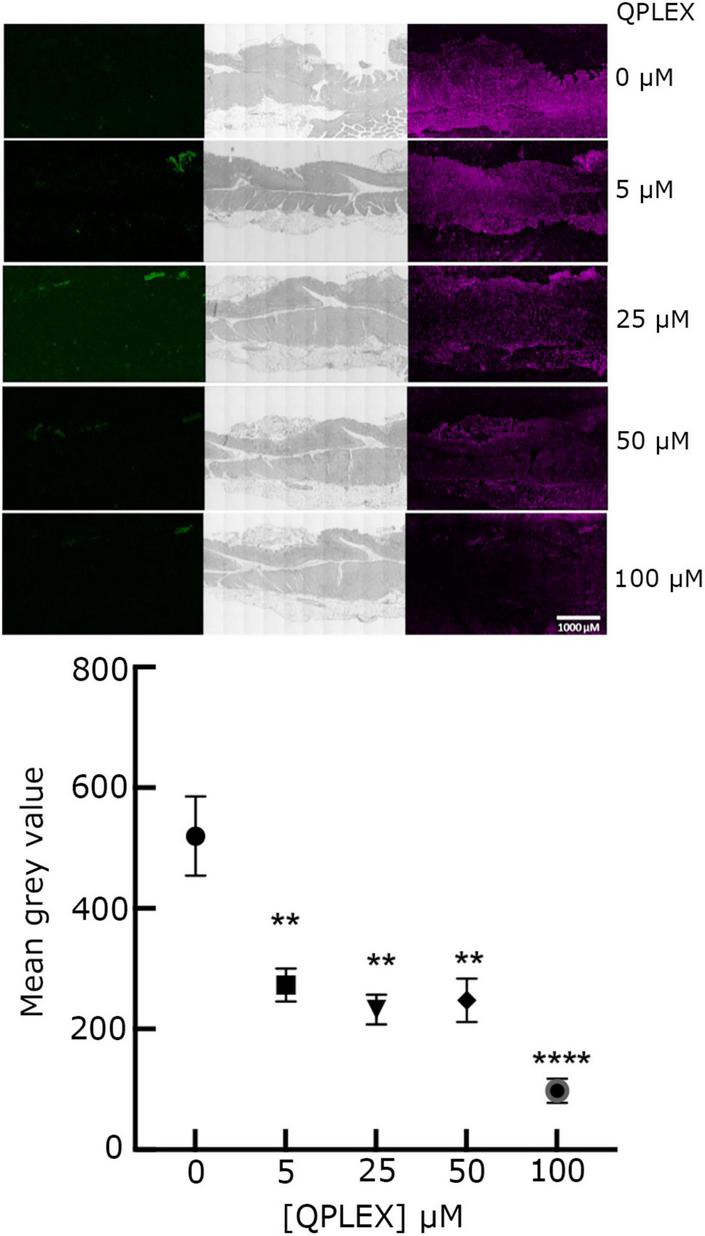
QPLEX inhibits binding of MOMP to Le^b^ + ve transgenic FVB/N mouse stomach tissue. The signal in the far-red channel was observed from binding of 5 μM MOMP incubated with increasing concentrations of QPLEX (0 to 100 μM) in a competitive binding study and then used to treat Le^b^ + ve transgenic FVB/N mouse stomach tissue slides. Staining followed by incubation with rabbit anti-MOMP primary antibody, then far-red tagged goat anti-rabbit antibody (right column). Fluorescence in the green channel was also examined to show the lack of background signal post quenching with autofluorescence quencher (left column). Light phase images show detailed structures of each tissue slide (middle column). The slide images shown are representative of a larger set. Fluorescence quantification is reported as mean gray values of total fluorescence corrected for background fluorescence observed at each concentration of QPLEX staining with standard error bars about the mean of 20 slide replicates per QPLEX concentration. ANOVA tests were used to assess the significance of differences between means in non-inhibited binding and inhibition analyses (** = *p* < 0.05 and **** = *p* < 0.0005).

The effect of QPLEX on the binding of MOMP to Le^b^ was investigated *in vitro* with ELISA. Le^b^ (1 μM) was immobilized on the surface wells of ELISA plates and then solutions of MOMP, MOMP^T/G268^ and MOMP^Δ pseD^ proteins (1 p.m.) were added to the wells and left to bind for 16 h in the absence and presence of QPLEX (1 nM). The results showed that QPLEX significantly inhibited the binding of native MOMP to Le^b^ (Figure 5A). As expected, binding of MOMP^T/G268^ and MOMP^Δ pseD^ to Le^b^ was much reduced compared to native MOMP and the presence of QPLEX made no significance difference (Figure 5A). This is consistent with previous work indicating that removal of the *O*-glycosylation at the T268 site significantly reduces the ability of MOMP to bind to BgAgs, including Le^b^ (Mahdavi et al., 2014). Furthermore, ELISA assays using digoxigenin tagged *C. jejuni* NCTC11168 bacteria in the presence or absence of QPLEX showed that in the presence of QPLEX (34 μM) the ability of the bacteria to adhere to BgAgs, including Le^b^, was substantially inhibited (Figure 5B). The competitive inhibitory effect of MOMP binding to Le^b^ was also confirmed with *ex vivo* immunofluorescence imaging of tissue slices (Figure 6).

Collectively, our *ex vivo* and *in vitro* data show that (i) MOMP binds to BgAgs, including Le^b^, (ii) glycosylation of MOMP at T268 is important for binding to BgAgs, including Le^b^ and (iii) QPLEX inhibits binding of MOMP to BgAgs, including Le^b^. The mechanistic details of this inhibition are not known but QPLEX has been shown to bind to MOMP and enter through the porin channel into the periplasmic space (Mahdavi et al., 2014). Given these properties, the potential of QPLEX to be used as feed-additive to protect commercial broilers was assessed with *in vivo* trials.

### 3.3. QPLEX protects commercial broilers against *C. jejuni* infection

The zootechnical efficacy of QPLEX in broilers was assessed with a detailed study at the Roslin Institute (see Supplementary Information; Supplementary Tables 1–9). Briefly, 420 males (breed/strain: Ross 308), day-old broilers were randomly allocated to 12 replicate pens of 35 broilers/pen. Two dietary treatments were allocated to pen replicates so that each treatment was applied to 6 pens: T1 Control and T5 QPLEX-treated (0.22 g/kg). To test QPLEX in broiler farm conditions, a natural infection mode using *Campylobacter spp.* infected litter was chosen over the more artificial challenge infection mode where broilers are orally inoculated with a single species of *C. jejuni*. Birds were fed from day 0 to 42 consecutively in mash form. The nutrient composition of all experimental diets was within the expected range with no notable differences between treatments. General health was good with low mortality/culls and no significant difference between the two dietary treatments, 2.9% in the T1 Control and 2.4% in the T5 QPLEX-treated groups (Supplementary Table 9). Prior to slaughter at day 42, a period of 16 h feed withdrawal was applied.

**FIGURE 7:**
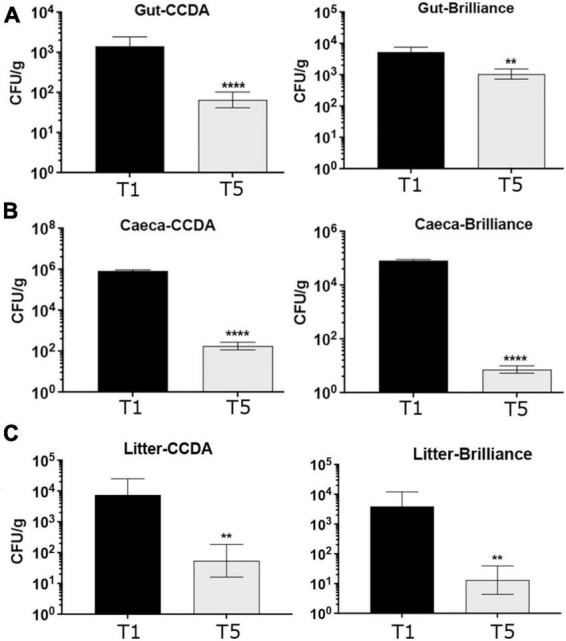
Quantification of *Campylobacter spp*. from gut **(A)**, caeca **(B)**, and litter **(C)** broiler samples using CCDA and Brilliance agar plates, as indicated. The CFU/g in the T5 QPLEX-treated broiler group were significantly reduced in gut, caeca and litter samples compared to the T1 control group, quantified in both CCDA and brilliance agar plates (*p* = 0.0001, **p ≤ 0.01, and ****p ≤ 0.0001, *t*-test).

At that point, 2 birds/pen (6 pens and a total of 12 birds) from the control T1 group and 1 bird/pen (6 pens and a total 6 birds) from the QPLEX-treated T5 group were selected at random, euthanized and gut samples collected to give 18 samples in total. Determination and enumeration of *Campylobacter spp*. was carried out with microbiological analyses using *Campylobacter spp.* selective CCDA and Brilliance media. *Campylobacter spp*. selective blood agar CCDA is prepared from *Campylobacter* Blood-Free Selective Agar Base CM0739 and CCDA Selective Supplement SR0115 and can be used for the isolation of *C. jejuni*, *Campylobacter coli*, and *Campylobacter laridis*. *Campylobacter spp*. chromogenic Brilliance CampyCount agar is a medium specifically designed for accurate, specific and easy enumeration of *C. jejuni* and *C. coli* from poultry. It is a transparent medium on which *Campylobacter* produces distinct dark red colonies, making identification and counting significantly easier than on traditional charcoal or blood-containing agar. Additional confirmatory tests were also carried to confirm lack of growth aerobically and growth micro-aerobically, indicative of *Campylobacter spp*., as well as oxidase positive tests to further confirm the presence of *Campylobacter spp*. The overwhelming majority of colonies (∼99%) were *Campylobacter spp* but occasionally very few colony morphotypes were suspected to be contaminants by their appearance in all pens, including the control T1 group (Supplementary Figure 5A). Further verification of *Campylobacter spp* as well identification of the distribution of *C. jejuni* versus *C. coli* was carried out by multiplex PCR, using primer sets that target the identification of *C. jejuni* and *C. coli*, based on amplification of two genes, *mapA C. jejuni* (589 bp) and *ceuE C. coli* (462 bp) (Begum et al., 2015). In addition, a 16S primer (800 bp) set was included as quality assurance for the DNA-preparation and analysis (internal control). Between 3 and 4 colonies from each treatment group were examined and all colonies were identified as *C. jejuni*. Supplementary Figure 5B gives an example of 3–4 representative colonies from various treatment groups examined. Our collective data show that *Campylobacter spp*. loads were reduced significantly reduced (1 × log_10_ reduction) in the T5 group gut samples compared to the T1 group gut samples (Figure 7A).

To analyses caeca samples at day 42 in a similar manner to the gut samples, 5 birds/pen were randomly selected and euthanized. Caeca samples were taken (1 pair caeca/bag) and quantified/enumerated for *Campylobacter spp*. The number of Colony Forming Units (CFU) of *Campylobacter spp*. were based on 5 replicas from each pen (6 pens and a total of 30 birds in total from each of the T1 and T5 groups) using CCDA and Brilliance media. Even bigger reductions (4 × log_10_) were observed in the *Campylobacter spp*. loads in caeca samples in the QPLEX-treated T5 group compared to the control T1 group (Figure 7B).

To assess possible effects of QPLEX on the growth performance of the broilers, the mean AWG (average weight gain) per pen, AFI (average feed intake) per pen and the feed conversion ratio (FCR, feed: gain) zootechnical parameters were calculated for the periods 1–21, 21–41, and 1–41 days on trial (Supplementary Tables 10–12). QPLEX did not adversely affect any of these zootechnical parameters. On the contrary, all of these parameters were either the same or exhibited a near significantly better trend compared in the T5 QPLEX group compared to the T1 control group.

Finally, at the end of the broiler trial litter samples were collected from each of the 6 pens from the T1 and T5 groups to be tested for *Campylobacter spp.* loads using selective CCDA and Brilliance media. Interestingly, litter from the T5 group showed a significant (2 × log_10_) reduction in *Campylobacter spp.* loads compared to the T1 group (Figure 7C).

## 4. Discussion

Ferric quinate is a complex of ferric ion and three quinic acid ligands (Supplementary Figure 6). D-(-)-quinic acid is essential to promote metal ion uptake in useable, soluble form in plants (Menelaou et al., 2009). QPLEX is also used as ferric source in magnetotactic bacteria, resulting in better growth (Yang et al., 2001). Furthermore, Ferric tyrosinate (TYPLEX), a complex of ferric ion with three tyrosinates and a structural analogue of QPLEX, can reduce *C. jejuni* loads in broiler farming, demonstrating the potential beneficial use of such iron chelates as feed additives in animal husbandry (Currie et al., 2018; Khattak et al., 2018; Skoufos et al., 2019). As the use of preventative antibiotics in animal feeds has contributed to the spread of antibiotic resistance globally, there is a pressing need to find novel antibiotic-free alternatives for use in animal farming. *C. jejuni* infection is a key global cause of gastrointestinal disease in humans with the incidence of the disease on the increase in the past 2 decades (Kaakoush et al., 2015). Many human infections (50–58%) can be traced to infected chickens with 20–30% of cases accounted for from mishandling, preparing and consuming of broiler chickens (Panel, 2011). MOMP is one of the major adhesins that mediates the attachment of *C. jejuni* to the gastrointestinal epithelium to establish infection and induce the release of proinflammatory cytokines (McSweegan and Walker, 1986; Hickey et al., 2000). In fact, a single T268G mutation in MOMP abolishes the ability of *C. jejuni* to establish infection in chickens (Mahdavi et al., 2014). Protein-glycan interactions on the surfaces of gastrointestinal epithelia and *C. jejuni* mediate bacteria adherence (Day et al., 2015). *O*-glycosylation of NCTC11168 *C. jejuni* MOMP seems to be important for mediating interactions with glycans, including Le^b^ (Mahdavi et al., 2014). Le^b^ was also detected in the adherence mucus layer of HT29-MTX-E12 human cells acting as receptors for *C. jejuni* binding and subsequent colonization (Naughton et al., 2013). Glycan array studies showed that human and chicken *C. jejuni* isolates bind to many different glycans with chicken isolates exhibiting a broader range of glycan binding compared to human isolates (Day et al., 2013). These wide range interactions of *Campylobacter spp* BgAgs contrasts the high degree of specificity exhibited by its close relative, *H. pylori* (Ilver et al., 1998; Mahdavi et al., 2002). This likely reflects the restricted host range of *H. pylori* (infecting only humans/primates) whereas *C. jejuni* can establish infection in a wide range of birds and mammals, gaining an evolutionary advantage by broadening its specificity and maximizing its survival in different hosts. Therefore, inhibiting MOMP-mediating interactions between *C. jejuni* and host epithelia will be key to preventing Campylobacteriosis.

Here, we show that QPLEX competitively inhibits binding of MOMP to the Le^b^ antigen on the gastrointestinal epithelia and substantially reduces *Campylobacter spp*. loads in commercial broilers when used as animal feed additive. We also confirm the importance of *O*-glycosylation of MOMP at T268 for adherence to gastrointestinal epithelia. Using *ex vivo* experiments with gastrointestinal tissue sections from the FVB/N transgenic mice that express Le^b^, we demonstrated specific binding of MOMP to Le^b^ and QPLEX-mediated inhibition of this interaction. We reinforced our *ex vivo* data with *in vitro* ELISA and confirmed the importance of MOMP *O*-glycosylation at T268 for binding to Le^b^. We demonstrated competitive inhibition of Le^b^ binding to MOMP by QPLEX with *ex vivo* immunofluorescence imaging and *in vitro* ELISA assays. The efficacy of QPLEX as an inhibitor of *Campylobacter spp* colonization in chickens was assessed and confirmed with *in vivo* trials in commercial broilers. The use of QPLEX as a feed additive at 0.22 g/kg reduced *Campylobacter spp.* loads significantly, by 1 × log_10_ in gut samples and by 4 × log_10_ in caeca samples. Furthermore, litter from QPLEX treated chickens exhibited a 2 × log_10_ reduction in *Campylobacter spp.* loads. It remains to be investigated whether higher concentrations of QPLEX and/or a combination of TYPLEX/QPLEX might result in even bigger reductions in *Campylobacter spp.* loads. These data are consistent with similar results obtained from the use of ferric tyrosinate (TYPLEX) (Currie et al., 2018; Khattak et al., 2018; Skoufos et al., 2019), demonstrating that such compounds can be used in broiler feed as alternatives to preventative antibiotics to combat *Campylobacter spp* infections in broiler farming. Furthermore, we present additional evidence here that these compounds specifically and exclusively prevent the attachment of *Campylobacter spp* to epithelial glycans of broilers, although we cannot exclude the possibility that they may also exert additional effects particularly as QPLEX has been shown to enter into the periplasmic space (Okoye et al., 2022). Furthermore, they do not exhibit any bactericidal or bacteriostatic activities and they are therefore unlikely to encourage long-term resistance. The stereochemical fit of the molecular interactions between microbial adhesion proteins and host tissue receptors determine in part the tissue tropism-directed colonization process. Interfering with adhesin-receptor recognition provides a new approach for the development of antimicrobial strategies. Receptor analogues have already demonstrated efficient anti-inflammatory therapies by reducing adherence of inflammatory cells to the endothelial cell lining, preventing recruitment of leukocytes to the area of inflammation thus reducing injury caused by inflammation (Mulligan et al., 2000). Therefore, ferric chelate analogues may provide a new approach for preventing *Campylobacter spp* infections in poultry which will also help to eliminate the use of preventative antibiotics thus contributing toward limiting the spread of antibiotic resistance through the broiler farming industry.

## Data availability statement

The original contributions presented in this study are included in the article/Supplementary material, further inquiries can be directed to the corresponding authors.

## Ethics statement

The animal study was reviewed and approved by the Roslin Institute.

## Author contributions

JCO: investigation, data curation, formal analysis, and review and editing. MP, VP, and OD: investigation and data curation. AP: methodology. TB: review and editing. NO and JM: supervision and review and editing. PS: funding acquisition, project administration, resources, supervision, data curation, formal analysis, writing—original draft, and writing—review and editing. All authors gave final approval for publication and agreed to be held accountable for the work performed therein.
