# Supplementary material for: Ferric quinate (QPLEX) inhibits the interaction of major outer membrane protein (MOMP) with the Lewis b (Leb) antigen and limits Campylobacter colonization in broilers
Source: Front Microbiol. 2023 Mar 10;14:1146418. doi: 10.3389/fmicb.2023.1146418 (PMC10036597; doi:10.3389/fmicb.2023.1146418)
Supplement: Supplementary file 1 [file Data_Sheet_1.PDF]

Ferric Quinate (QPLEX) inhibits the interaction of Major Outer Membrane Protein (MOMP) with the Lewis b (Le^b) antigen and limits Campylobacter colonisation in broilers

Jennifer C. Okoye[1], Alexandria Holland[1], Matthaios Pitoulias[1], Vasileios Paschalis[1], Artem Piddubyi[3,4], Osman A. Dufailu[5], Thomas Borén[3], Neil J. Oldfield[2], Jafar Mahdavi[1,*], Panos Soultanas[1,*]

[1] School of Chemistry, Biodiscovery Institute

University of Nottingham, University Park

Nottingham NG7 2RD, UK

[2] School of Life Sciences , University of Nottingham

University Park
Nottingham , NG7 2RD , UK

[3] Dept. Medical Biochemistry and Biophysics
Umeå University, 901 87, Umeå , Sweden

[4] SUMEYA, The Ukrainian-Swedish Research Center,
Sumy State University, 40022 Sumy, Ukraine

[5] School of Science, Faculty of Engineering & Science

Medway Campus, University of Greenwich, UK

*Corresponding author: panos.soultanas@nottingham.ac.uk, jafar.mahdavi@outlook.com

SUPPLEMENTARY INFORMATION

Optimisation of *ex vivo* analyses to eliminate background fluorescence

Human tissue sections from the upper (corpus) and lower (antrum) parts of the stomach, as well the duodenum and colon of the gut, were visualised in the green fluorescence channel (487 mm) to assess the extent of background auto-fluorescence and the potential use of $MOMP^{flu}$ for further imaging. The images revealed significant auto-fluorescence in the green spectrum making the use of $MOMP^{flu}$ problematic although detailed structural characteristics of the different tissues were also revealed (Figure S1). A spectral scan then was carried out to ascertain the extend of background auto-fluorescence in the blue, green and far-red spectra to help us ascertain the use of an appropriate probe in our imaging studies. Images revealed that there was background auto-fluorescence in all three channels but the far-red channel exhibited the lowest background auto-fluorescence, indicating that using the far-red channel will be better for our downstream fluorescence imaging experiments (Figure S2). To decrease the background auto-fluorescence further, the Vector® TrueVIEWTM Autofluorescence Quenching Kit was used (Karpishin, 2018), resulting in excellent reduction of background autofluorescence with no structures of the tissue being visible at pre-quench levels of brightness and contrast (Figure S3).

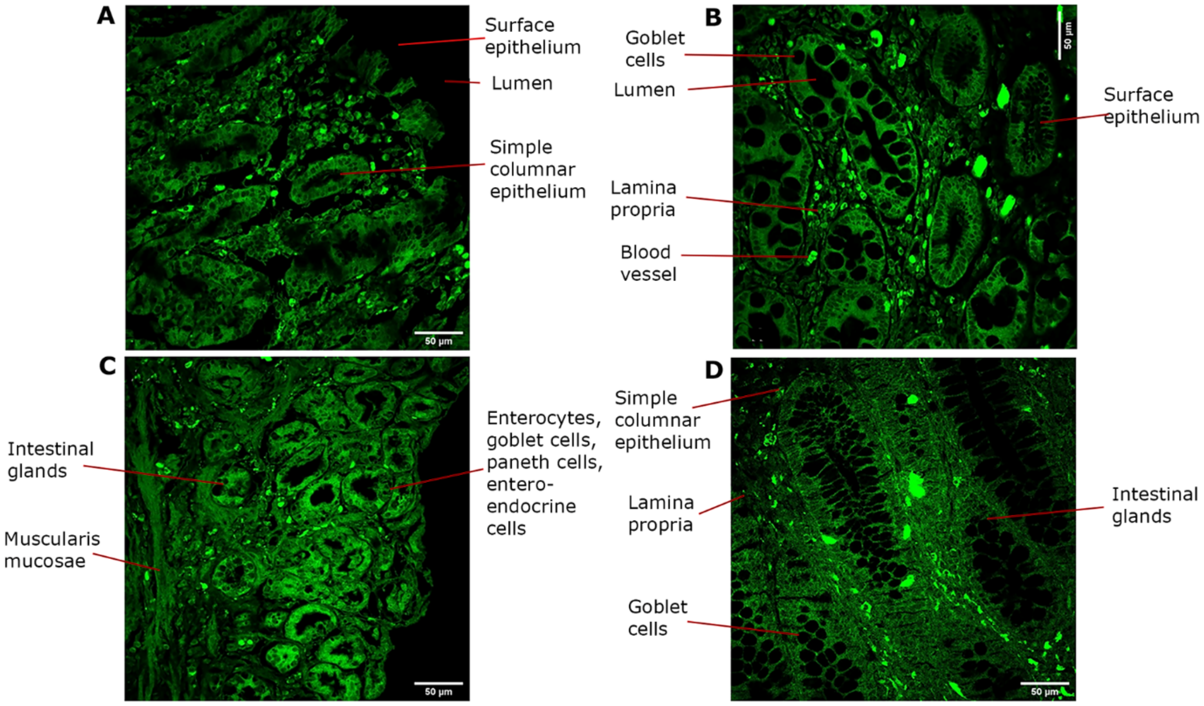

Figure S1: Images of unstained sections of corpus (A), antrum (B), duodenum (C) and colon (D) taken at 487 nm. Brightness and contrast have been increased to show structural detail. Scale bars represent 50 µm. In the corpus we see the thick mucosa lined with simple columnar epithelium that invaginates into gastric pits into which the fundus glands open. In the antrum, we see the same general structure as in the corpus with epithelial lined villous folds that invaginate into gastric pits. The cells lining the villous folds are surface mucus cells that produce alkaline mucus to protect the gastric mucosa from the acidic content of the stomach. In the duodenum the auto-fluorescence highlights intestinal glands (aka crypts of Lieberkeun) which are lined with numerous relatively undifferentiated columnar cells that usually undergo two rounds of mitosis before differentiating into either absorptive cells or goblet cells. The cells that line the intestinal glands are enterocytes, goblet cells, paneth cells and enteroendocrine cells. Usually, eosinophilic granules of paneth cells lie within the apical cytoplasm of the intestinal glands, as they recognise antibacterial enzymes secreted from the paneth cells. At the edge of the image is the muscularis mucosae, a thin layer of smooth muscle that marks the bottom end of the mucosa. In the colon we see simple columnar epithelium on the edge of the lumen where the surface columnar epithelium and the cells lining the crypts are enterocytes, with an oval basal nucleus and apical brush border, the microscopic representation of microvilli. Simple tubular intestinal glands (crypts of Lieberkuhn) extend through the entire thickness of the mucosa. There are also numerous mucous secreting goblet cells recognized by their content of a large mucous globule. The lamina propria with connective tissue and inflammatory cells surround the crypts.

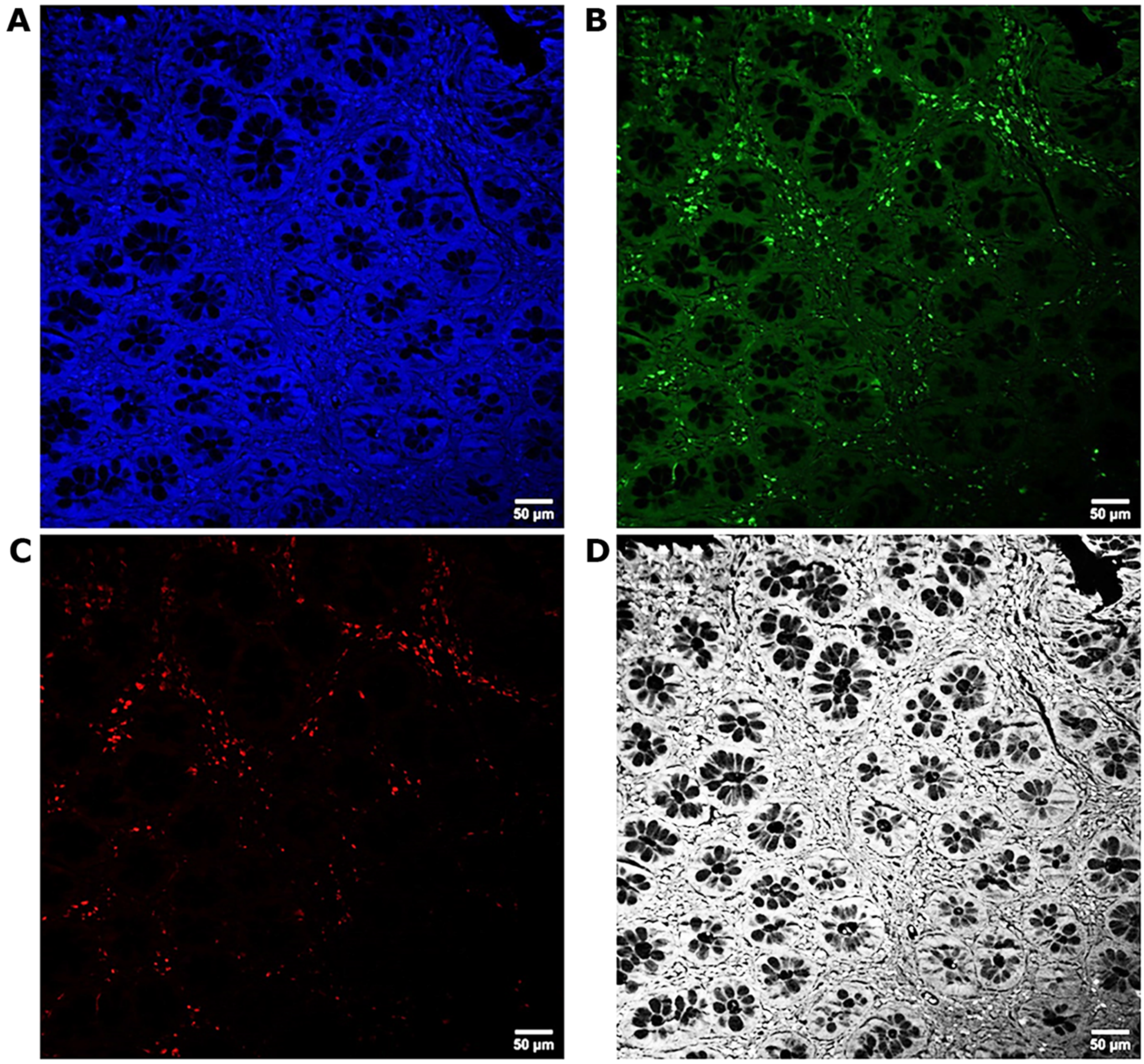

Figure S2: Images of the human stomach corpus were captured at wavelengths in the blue (**A**), green (**B**) and far-red (**C**) channels during a spectral scan. Digitally inverted white light image for structural detail is also shown in panel D. The minimum and maximum brightness and contrast levels are consistent to portray different levels of fluorescence from the same tissue section. Scale bars indicate 50 µm. Histogram visualisation settings: Minimum 274, maximum 6185.

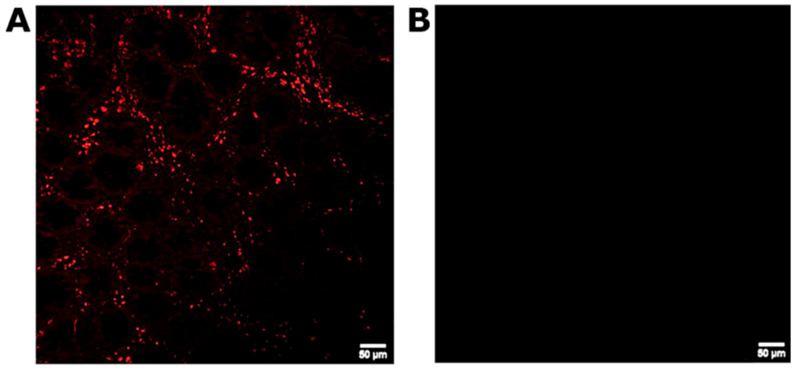

Figure S3: Fluorescence images the human corpus in the far-red fluorescent channel after treating the tissue with Vector® TrueVIEWTM autofluorescence quencher**.** Background auto-fluorescence before quenching **A** and after quenching **B**. Scale bar shows 50 μm length.

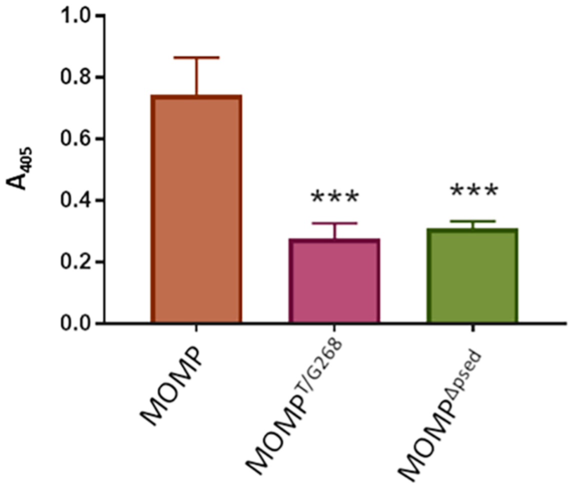

Figure S4: Binding of native MOMP, MOMP$^{T/G268}$ and MOMP$^{\Delta pseD}$ to Le^{b} in ELISA. Binding of all proteins (1 pM) was measured by incubating in amino linked ELISA wells coated with Le^{b} (1µM). This was followed by incubating with primary antibody against MOMP and alkaline phosphatase (AP) conjugated secondary antibody. The Tecan was used to measure absorbance at 405 nm by the AP substrate. The results show the maximum absorbance obtained during a 16 hours assay. BSA was used to reduce non-specific background binding and the measurements of BSA absorbance were subtracted from total absorbance of binding of each protein to Le^{b}. The bar graphs show the mean absorbance for each protein with standard error bars of the mean of triplicate data from three separate experiments. The statistical significant differences between Le^{b} binding to native MOMP, MOMP$^{T/G268}$ and MOMP$^{\Delta pseD}$ were analysed with ANOVA statistical analysis (***$p < 0.001$).

A

Other assays *Campylobacter spp,* Campy CCDA plate (Roslin study, RGT136/2016)								
Our ID	Treatment	Pen	No	Blood agar (MA)	Blood agar (A)	Oxidase	Gram stain	
13	T1 Gut	29	4	(+)	(-)	(-)	(+)	contamination
24	T1 Gut	11	1	(+)	(-)	(+)	(-)	
86	T1 Gut	6	5	(+)	(-)	(+)	(-)	
99	T1 Gut	25	3	(+)	(-)	(+)	(-)	
129	T1 Gut	3	3	(+)	(-)	(+)	(-)	
40	T5 Gut	32		(+)	(-)	(-)	(+)	contamination
82	T5 Gut	8	3	(+)	(-)	(+)	(-)	
87	T5 Gut	8	4	(+)	(-)	(+)	(-)	
100	T5 Gut	8	2	(+)	(-)	(+)	(-)	
114	T5 Gut	5	5	(+)	(-)	(+)	(-)	
157	T1 Caeca	27	1	(+)	(-)	(+)	(-)	
175	T1 Caeca	6	1	(+)	(-)	(+)	(-)	
195	T1 Caeca	27	3	(+)	(-)	(+)	(-)	
221	T1 Caeca	3	4	(+)	(-)	(+)	(-)	
275	T1 Caeca	27	5	(+)	(+)	(-)	(+)	contamination
170	T5 Caeca	5	1	(+)	(-)	(+)	(-)	
209	T5 Caeca	19	1	(+)	(-)	(+)	(-)	
219	T5 Caeca	15	1	(+)	(+)	(-)	(+)	contamination
252	T5 Caeca	5	2	(+)	(-)	(+)	(-)	
266	T5 Caeca	25	5	(+)	(-)	(+)	(-)	

MA: Microaerophilic condition
A: Aerophilic condition

B

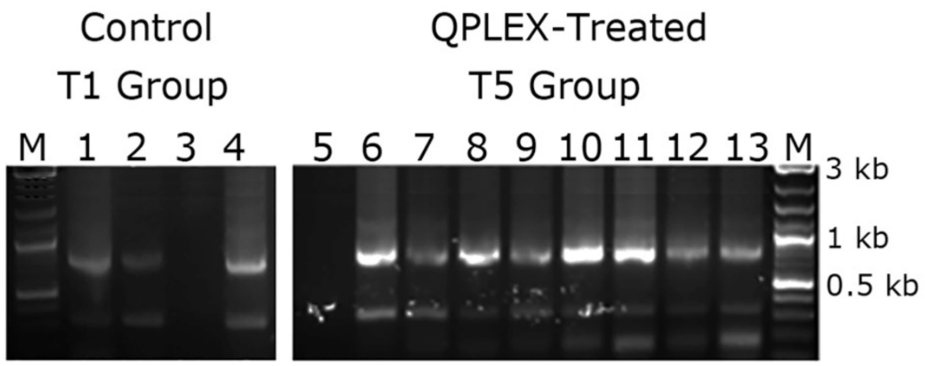

Figure S5: A. Detailed data from the identification of *Campylobacter spp* from various samples taken from different treatment groups. The overwhelming majority of the colonies examined were confirmed to be *Campylobacter spp* with only small numbers of contaminants. **B.** Multiplex PCR representative examples to confirm the distribution of *C. jejuni* versus *C. coli* and contaminants. The primer sets in this multiplex PCR target the identification of *Campylobacter jejuni* and *Campylobacter coli* based on the amplification of the two genes, *mapA* (589 bp) *ceuE* (462 bp). A 16S primer (800bp) was included as quality assurance of the DNA-preparation and analysis (internal control). Three different concentrations of each isolate's template were used for PCR amplification. Lanes 1-4 show representative samples from the control group T1 and lanes 5-13 show representative samples from the treatment group T5. All sample colonies from our CCDA and Brilliance agar plates selected for the PCR analysis were confirmed to be *C. jejuni* (see all lanes except lanes 3 and 5). A small number of colonies that looked suspiciously different were also tested by PCR to confirm their identity (see lanes 3 and 5 showing lack of amplification, hence not *Campylobacter spp*.

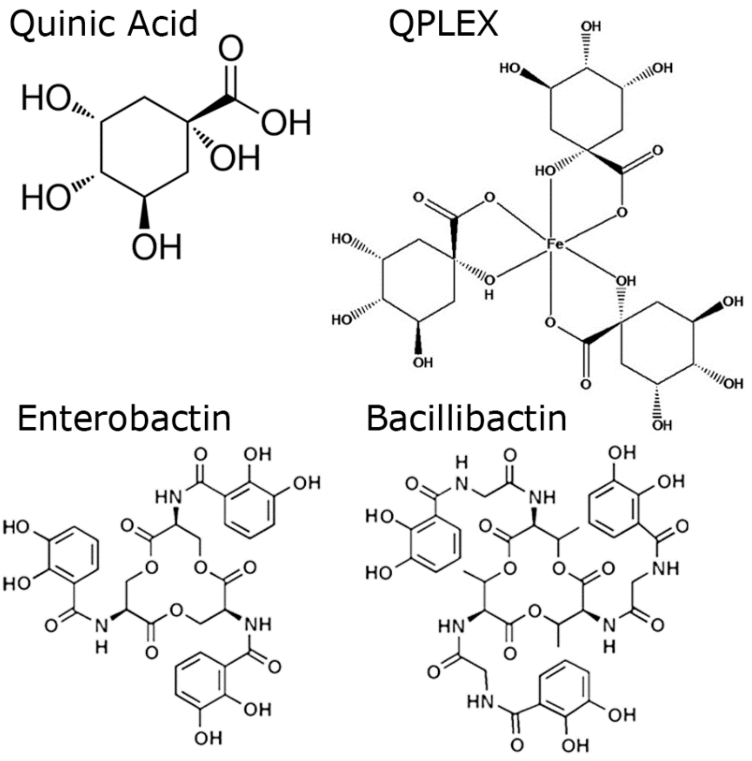

Figure S6: The structures of quinic acids, QPLEX and the bacterial siderophores enterobactin and bacillibactin. QPLEX is a structural mimic of bacterial siderophores.

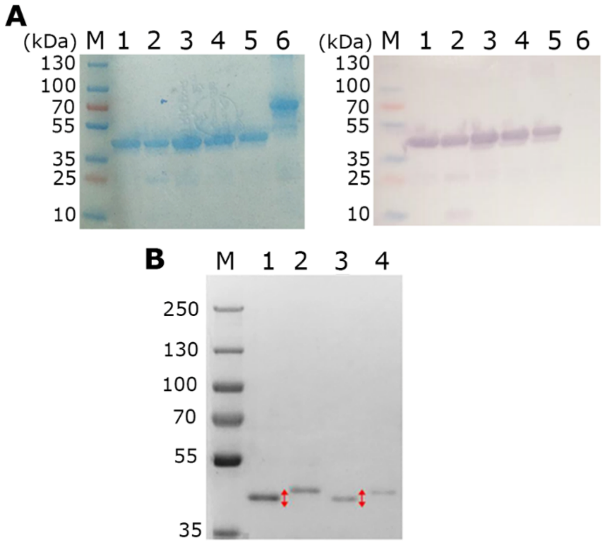

Figure S7: A. Western blot analysis of the wtMOMP (heterologously expressed and purified from *E. coli*), MOMP$^{T/G268}$ and MOMP$^{\Delta pseD}$ proteins. Proteins were resolved through a 10% SDS PAGE gel (left panel) and blotted onto nitrocellulose for a western blot (right panel) using rabbit anti-MOMP antibody. Lane M - ThermoFisher Prestained PageRuler Plus Stained Protein Ladder, lane 1- wt NCTC11168 MOMP, lane 2 – MOMP$^{T/G268}$, lane 3- MOMP$^{\Delta pseD}$, lane 4- MOMP^{flu} (MOMP conjugated to fluorescein), lane 5- MOMP^{farred} (MOMP conjugated to Alexa Fluor™ 647), lane 6 – control BSA. **B.** SDS PAGE of native NCTC11168 MOMP (lanes 2 and 4), MOMP$^{T/G268}$ (lane 1) and MOMP$^{\Delta pseD}$ (lane 3) proteins resolved through a 6% polyacrylamide gel. Visible migration differences are observed between the native MOMP and the MOMP$^{T/G268}$ and MOMP$^{\Delta pseD}$ proteins, as indicated by double-headed red arrows. Lane M shows a ThermoFisher Prestained PageRuler Plus Stained Protein Ladder.

Broiler study details at the Roslin Nutrition Ltd

Study location

Hen House 1, Fenton Barns, Roslin Nutrition Ltd., Gosford Estate EH32 0PX Aberlady Longniddry, East Lothian Scotland, UK.

Test products

Supplementary Table S1. Details of test products				
Products	Manufacturer	Lot Nº, Manufacture Expiry Date	Active substance	Guarantee
QPLEX	Akeso Biomedical Inc. USA	Lot: AMA-P-16-01-I-14<br>Made: 9/3/2016<br>Expiry: 8/3/2018	Fe quinic acid complex	As per CoA

Key study personnel

Study Director: Anne Knox, Gosford Estate EH32 OPX, Aberlady, Longniddry, East Lothian, Scotland, UK; Tel: +44 187 587 1270; E-mail: anne.knox@roslinnutrition.co.uk

Daily monitoring, animal care: Kenny Riding, Roslin Nutrition Ltd.

Veterinary Surgeon: Barry Thorp, St David's Poultry Team, Royal Dick Veterinary College, University of Edinburgh, Easter Bush Veterinary Centre, Midlothian EH25 9RG, Scotland.

Feed supplier, feed mill, supervision of diet manufacture: G. Dickson, Roslin Nutrition Ltd.

Feed proximate analysis: DM Scientific, UK.

Feed Fe analysis: Sciantec Analytical Services Ltd., Stockbridge Technology Centre, Cawood, North Yorkshire, YO8 3SD Direct: +44 (0) 1757 242414, Mobile: +44 (0)7917 064591

Feed mictrotracer-TYPLEX™/QPLEX™ analysis: Samples sent to USA, details as follows: David Eisenberg and Dr Vu Lam, Anresco Laboratory, 1375 Van Dyke Avenue, San Francisco, CA 94124, Zip Code 415-822-1100, Tel. E-mails: david@microtracers.com, vu@anresco.com Tel. +1-415-822-1100

Microbiology: Samples sent to the Biodiscovery Institute, University of Nottingham, University Park, Nottingham NG7 2RD United Kingdom. Tel: (44) 115 823 0749, Fax: (44) 115 823 0759, E-mail: jafar.mahdavi@nottingham.ac.uk

Experimental animals

Animals: Broilers, Breed/Strain: Ross 308 Origin: Commercial hatchery

Sex: Males | Physical status: Healthy | Initial age: 1-day-old
Final age: 42 days | Initial weight: ~40 g | Final weight: ~2 kg
Physiological status: Growing/fattening chicken

Initial health examination

Any chicks showing signs of ill health, injury or poor condition were excluded. Healthy day-old chicks (420) were distributed at random into 12 single-sex pens of 35 male birds/pen.

Treatment application

QPLEX (T5) were administered in the feeds as indicated in **Supplementary Table S2**.

Experimental treatments

Supplementary Table S2. Experimental treatments			
Treatment	Test product, g/kg feed	Nº replicate pens and birds	Microtraced test products g[1]/kg feed[2]
Control	Control - 0	6 pens x 35 birds/pen	0
QPLEX	Control + QPLEX (0.22 g/kg feed)	6 pens x 35 birds/pen	0.242[2]
Nº treatments: 2, Replicate pens/treatment: 6; Total nº of pen replicates: 12<br>Broilers/replicate: 35 (maximum EU stocking density); Broilers/treatment: 420;<br>[1]1 g of microtracer contains 60,000 red graphite particles.<br>[2]Microtracers at 10% in test products (Q-PLEX-red).			

Detailed study design

Supplementary Table S3. Study design	
Control	QPLEX
Broilers on trial from day-old to 42 days of age	
1-21 days of age	22-42 days of age
Starter feed	Grower feed
Observations:<br>Health check at 1 day of age.<br>Mean pen BW at 1, 21, 41 days of age, bird BW calculated<br>Mean pen AWG, AFI, FCR at/to 21, 41 days of age.<br>Caeca samples at 42 days sent for microbiology<br>Daily health records, mortality/culls, including reason for culls & probable cause of mortality.<br>All birds culled or dead were subjected to veterinary necropsy, if carcases fresh and causes not clear.<br>Adverse events were noted (*e.g.* power, feed, water failures, disease outbreak, *etc.*).	

General husbandry and management description

Pre-sexed, male day-old broilers were purchased from a local commercial source. Selected healthy broilers were allocated to 6 replicates (pens) of 35 broilers/pen. The 2 dietary treatments were allocated to pen replicates so that each treatment was applied to 6 pens in a RCB (random complete block) design. Birds were bedded on fresh wood shavings over used litter in a poultry barn. The house was lit by programmable artificial light. The standard lighting program was 23 hours of light per day, and 1-hour dark. Temperature inside the building was set as recommended by the breeder. Any chicks showing signs of ill-health, injury or of being in poor condition were excluded from the selection process. Mash diets were provided to the broilers *ad libitum* from feeders. Water was also provided *ad libitum* from bell drinkers. Environmental conditions during the trial (temperature and

ventilation rate) were automatically controlled and appropriate for the age of the broilers. The trial terminated after 42 days and all birds were culled and carcases destroyed.

Diet composition

Diets did not contain coccidiostats or veterinary antibiotics. For each feeding period (starter and grower), basal diets were calculated to be iso-nutritive, and to meet or exceed nutrient requirements recommended for broilers. The ingredients, premixes, the calculated and actual analyses of the diets are presented in **Supplementary Supplementary Table** S4 and **Supplementary Supplementary Table** S5.

Feed composition and calculated analyses

Supplementary Table S4. Composition and calculated analyses of diets

Ingredients (%)	Starter Mash<br>1-21 days of age	Grower Mash<br>22-42 days of age
Wheat	69.862	67.354
Barley	-	7.5
Soybean meal, 48% CP	23.4	21.4
Sodium bicarbonate	0.13	0.22
Fishmeal 66%	2.5	-
Soy oil	1.3	1.4
L-lysine HCl	0.128	0.175
DL-methionine	0.123	0.164
Choline chloride	0.067	0.067
Dicalcium phosphate	0.13	0.32
Calcium carbonate	1.74	0.74
Sodium chloride	0.12	0.16
Minerals and vitamins[1]	0.5	0.5
Total	100	100
Calculated analyses		
ME Broiler, MJ/kg	11.526	12.346
Crude protein %	21	19
Crude fibre, %	2.73	2.914
Ash, %	5.796	4.571
Dry matter, %	72.97	77.503
Crude fat %	3.0	3.0
Lysine	1.18	1.050
Methionine	0.45	0.438
Methionine + cystine	0.797	0.766
Threonine	0.75	0.661
Tryptophan	0.259	0.237
Calcium	1.102	0.651
Sodium	0.126	0.142

[1]Supplies per kg: Vit A: 12,000 IU; Vit D_3: 2,400 IU; Vit E: 30 mg; Vit K_3: 3 mg; Vit B_1: 2.2 mg; Vit B_2: 8 mg; Vit B_6: 5 mg; Vit B_{12}: 11 µg; Folic acid: 1.5 mg; Biotin: 150 µg; Ca pantothenate: 25 mg; nicotinic acid: 65 mg; Ethoxyquin: 150 mg; Mn: 60 mg; Zn: 40 mg; I: 0.33 mg; Fe: 80 mg; Cu: 8 mg; Se: 0.15 mg.

Feed analyses

Proximate nutrients, Fe and microtraced test products were determined in feeds prior to study start to confirm correct diet mixing, content of test products and key nutrients (**Supplementary Table S5**).

Supplementary Table S5. Analysed values of experimental diets								
Sample Date	Sample	Dose, g/kg	Moisture (%)	Crude protein (%)	Ether extract (%)	Ash (%)	Fe (mg/kg)	Test product-microtracer (% recovery*)
10/8/2016	T1 starter	0	11.6	20.4	3.1	5.5	125	0
10/8/2016	T5 starter	0.22	11.7	20.5	2.8	5.4	150	72
10/8/2016	T1 grower	0	11.9	19.1	3.2	4.2	171	0
10/8/2016	T5 grower	0.22	12.0	18.8	2.9	4.5	129	92
*Refer to Appendix 3 for more details								

Feed/premixture manufacture

The experimental diets were formulated by Roslin Nutrition Ltd., who also provided the basic ingredients for the diets. All diets were mixed using a horizontal ribbon blade mixer. This mixer had a capacity of 1,000 kg and all diets were mixed for a period of 3 minutes. Feeds were bagged into 20 kg, 3-ply Kraft brown paper sacks, each of which was uniquely labelled with the study name (TYPLEX efficacy in broilers), unique study code (RGT136/2016), and treatment code (T1 and T5, as relevant), after which the diets were stored on labelled pallets in a dry, cool place ready for dispatch to the farm. First, QPLEX (for QPLEX diets) were premixed into 1 kg of wheat, then into 20 kg of wheat before being added and mixed into the final diet, which was then bagged. All diets were prepared without the inclusion of any coccidiostats or veterinary antibiotics (**Supplementary Supplementary Table** S4) and were analysed for crude protein, ether extract, dry matter, Fe, ash and microtraced test products (**Supplementary Table S5**). The results of these analyses acted as a double check on feed homogeneity. Separate feed batches were prepared for each dietary treatment. The approximate total amounts of the diet required for the study was 4 tonnes including 20% safety margin = (2 tonnes per treatment). Akeso Medical Inc. USA provided the appropriate quantity of QPLEX (555 g microtraced Q-PLEX). All feed remaining after the trial was accounted and properly disposed. Unused samples of QPLEX were returned to the study sponsor.

Feeding system

One bag during use was placed outside each pen, which was uniquely identified with the treatment and replicate number. Broilers on trial had *ad libitum* access to mash feed and water.

Feed samples and analysis at manufacture

From each feed batch (control and QPLEX), Roslin sent 200 g samples to Sciantec for checking Fe; 200 g samples to DM Scientific for proximate analysis; 2 x 1 kg samples for microtracer analysis and for storage frozen at Roslin until issue of study final report; and 1 x 500 g samples for storage frozen for transporting to the University of Nottingham. Feed samples sent to the University of Nottingham were labelled with the unique study code (RGT136/2016), the treatment code (T1 and T5, as relevant), the type of diet (e.g. mash starter or mash grower), the date of manufacture and the analysis required (proximate, Fe, microtraced test products). Please also refer to **Supplementary Table S7** for feed sampling and labelling instructions.

Supplementary Table S6. Feed sampling plan		
Treatment	Starter	Grower
	Mash, at manufacture	Mash, at manufacture
T1 Control	2 × 1 kg, 2 × 200 g, 1 x 500 g	2 × 1 kg, 2 × 200 g, 1 x 500 g
T5 QPLEX	2 × 1 kg, 2 × 200 g, 1 x 500 g	2 × 1 kg, 2 × 200 g, 1 x 500 g
1 x 200 g T1 and T5 to DM Scientific for proximate analysis<br>1 x 200 g T1 and T5 to Sciantec for Fe analysis<br>1 x 1 kg T1 and T5 to Anresco USA for microtracer analysis*<br>1 x 500 g to Dr Jafar Mahdavi for storage frozen<br>1 x 1 kg T1 and T5 stored frozen at Roslin as back-up samples		
*Samples destined for the University of Nottingham were labelled as: T1, T5,<br>"Research Samples – Do not Contain Animal-Derived Substances or Animal By-Products"		

Supplementary Table S7. Feed sampling labelling				
Label	Feed Samples ("Research Samples" for USA-destined samples*)			
Study Identification	Efficacy of QPLEX in Broilers			
Unique Study Code	RGT136/2016			
Treatment Code Number	T1, T5			
Type of sample	Starter	Grower		
Presentation	Mash			
Date of manufacture:	10 August 2016			
Date of sampling:	10 August 2016			
Bag nº				
Analyses	Proximate: crude protein, fat, ash, moisture	Iron (Fe)	Store frozen at RNL & sent to Dr Jafar Mahdavi, Nottingham University	Microtraced test products* Akeso & Anresco advised of sample shipping in advance
*Samples destined for the University of Nottingham were labelled as: T1 and T5.<br>"Research Samples – Do not Contain Animal-Derived Substances or Animal By-Products"				

Description of parameters recorded

* Health evaluation of all birds at 1 day of age. Any poor chicks excluded.

* Pen BW (body weight) at 1, 21 and 41 days of age, individual bird BW calculated.

* Mean pen AWG (mean weight gain), AFI (mean feed intake) and feed efficiency (FCR, feed:gain) calculated for periods 1-21, 21-41 and 1-41 of days on trial.

* Daily health records, mortality/culls, including reason and probable cause of culls/mortality.

*Any adverse events noted (*e.g.* power failure, feed/water failures, disease outbreak, *etc*.).

* Feed was withdrawn at 5.00 pm the day before study end, but birds had access to drinking water. At study end (42 days), five birds per pen, selected at random were euthanized. Caeca were removed intact and were immediately frozen on dry ice, then sent for microbiological analyses at the University of Nottingham (**Supplementary Table S8**). Samples were sent on dry ice via World Courier, monitored express service.

Supplementary Table S8. Caeca samples at study end			
Caeca samples	42 d	5 animals selected at random from each pen were euthanized and caeca samples collected to give 61 samples in total (included 1 extra sample from treatment group 5). Both caeca were tied off & bagged.	Caecal samples were frozen immediately on dry ice & sent for *Campylobacter jejuni* counts. Caecal samples were packed individually: 1 pair caeca/bag, labelled as caecal samples, with treatment code, pen nº & bird nº (birds 1-5)

Measures taken to avoid cross contamination between groups

All T1 control feeds were made before T5 QPLEX feeds.

Supplementary Table S9. Mortality, culls, and causes of removal from study							
Treatment	Pen nº	Date	Weight (kg)	Death or Cull	Cause of death/cull	Nº birds/treatment	%
T1 Control	3	12/10/16	0.80	C	Off legs	6	2.9
	6	11/10/16	0.32	C	Small bird		
		19/10/16	0.40	C	Small bird		
	27	23/10/16	1.50	D	Not determined		
		24/10/16	1.04	C	Off legs		
	29	17/10/16	0.40	C	Small bird		
T5 QPLEX	8	30/09/16	0.10	C	Small bird	5	2.4
	25	30/09/16	0.10	C	Small bird		
		17/10/16	0.56	C	Small bird		
	19	13/10/16	0.18	C	Small bird		
		18/10/16	0.46	C	Small bird		
Notes: C = culled, D = died							

Measurements of zootechnical parameters

Q-PLEX™ did not affect any zootechnical parameter in the overall study period.

Supplementary Table S10. Zootechnical performance from 1 to 21 days of age (d)						
Treatment	QPLEX Dose g/kg	BW, 1 d g	BW, 21 d g	AWG g	AFI g	FCR feed:gain
T1 Control	0	41.8	520	478	737	1.539
T5 Q-PLEX™	0.22	42.2	547	505	772	1.530
Nº replicates/treatment = 6 pens of 35 male birds/treatment; Means separated by Tukey Test.<br>BW = mean bird weight; AWG = average weight gain per pen; AFI = average feed intake per pen; FCR = feed/gain.						

Supplementary Table S11. Zootechnical performance from 21 to 41 days of age (d)						
Treatment	QPLEXD ose g/kg	BW, 21 d g	BW, 42 d g	AWG g	AFI g	FCR feed:gain
T1 Control	0	520	1,868	1,348	2,488	1.845
T5 Q-PLEX™	0.22	547	1,900	1,353	2,528	1.871
Nº replicates/treatment = 6 pens of 35 male birds/treatment; Means separated by Tukey Test.<br>BW = mean bird weight; AWG = average weight gain per pen; AFI = average feed intake per pen; FCR = feed/gain.						

Supplementary Table S12. Zootechnical performance from 1 to 41 days of age (d)						
Treatment	QPLEX Dose g/kg	BW, 1 d g	BW, 42 d g	AWG g	AFI g	FCR feed:gain

T1 Control	0	41.8	1,868	1,827	3,224	1.765
T5 Q-PLEX™	0.22	42.2	1,900	1,858	3,301	1.778
Nº replicates/treatment = 6 pens of 35 male birds/treatment; Means separated by Tukey Test.<br>BW = mean bird weight; AWG = average weight gain per pen; AFI = average feed intake per pen; FCR = feed/gain.						

REFERENCES

Karpishin T. 2018 Reducing Tissue Autofluorescence. *Biotechniques*, **64**, 131.

AOAC, 2000 Official Methods of Analysis (17th Ed.) Association of Official Analytical Chemists, Arlington, VA, USA.

Commission Regulation (EC) Nº 429/2008 of 25 April 2008 on detailed rules for the implementation of Regulation (EC) Nº 1831/2003 of the European Parliament and of the Council as regards the preparation and the presentation of applications and the assessment and the authorisations of feed additives. http://eur-lex.europa.eu/en/index.htm.

EFSA (2014) Technical and administrative guidance documents in relation to feed additive authorisations in the EU, last updated September 2014, http://www.efsa.europa.eu/.

Regulation (EC) Nº 1831/2003 of the European Parliament and of the Council of 22 September 2003 on additives for use in animal nutrition. http://eur-lex.europa.eu/en/index.htm.

NRC, 1994. Nutrient Requirements of Poultry. 10th Rev. Ed. National Academy Press. Washington DC. USA.
